# Type 2 Myocardial Infarction: Navigating Diagnostic Pathways and Therapeutic Crossroads Between Invasive and Conservative Strategies

**DOI:** 10.3390/jcm15031279

**Published:** 2026-02-05

**Authors:** Sebastian Cinconze, Chiara Bernelli, Francesca Giordana

**Affiliations:** 1 Division of Cardiology, Ospedale Civile di Vigevano, 27029 Vigevano, Italy; 2 Cardiology Unit, Santa Corona Hospital, ASL2 Liguria, Via XXV Aprile 38, 17027 Pietra Ligure, Italy; 3 Division of Cardiology, Ospedale Santa Croce e Carle, 12100 Cuneo, Italy

**Keywords:** myocardial infarction, coronary angiography, medical therapy

## Abstract

Type 2 myocardial infarction (T2MI) is defined as myocardial necrosis caused by an imbalance between oxygen supply and demand in the absence of acute atherothrombotic coronary occlusion/erosion. Unlike type 1 myocardial infarction (T1MI), T2MI comprises a heterogeneous group of clinical scenarios, often triggered by systemic or cardiac conditions, and it frequently affects elderly patients with a high burden of comorbidities. T2MI often underline multivessel coronary artery disease and, despite its growing clinical relevance, the diagnostic and therapeutic approach to T2MI remains challenging and lacks standardized recommendations. In this review, we present an updated and a comprehensive synthesis of current evidence on the diagnosis and management of T2MI, focusing on the role of coronary angiography and interventional strategies. We discuss the utility of high-sensitivity cardiac biomarkers, imaging modalities, and clinical risk scores to guide patient selection for invasive evaluation. Specific attention is given to conservative and alternative revascularization approaches—including drug-coated balloon angioplasty and stentless percutaneous coronary intervention (PCI)—in frail and high-bleeding-risk patients. The review emphasizes the need for individualized decision-making in a population where standard invasive strategies may not always be appropriate, and where a tailored balance between ischemic and hemorrhagic risk is crucial.

## 1. Introduction

According to the Global Burden of Disease Study, ischemic heart disease remains one of the leading causes of mortality globally [1]. Over recent decades, interventional management of acute myocardial infarction (AMI) has advanced substantially; however, rates of short- and mid-term morbidity and mortality remain significant [2,3,4]. While there is broad consensus regarding the optimal treatment of Type 1 AMI, considerable uncertainty persists in the diagnosis, clinical management, and therapeutic strategies for Type 2 AMI [5]. This ambiguity stems from the heterogeneous clinical scenarios underlying Type 2 AMI, the complexity and frailty of affected patients, and the paucity of robust evidence in the literature [6,7]. This review aims to synthesize and clarify the current evidence base for this clinical setting.

## 2. Epidemiology and Clinical Profile of Type 2 Myocardial Infarction

Type 2 myocardial infarction (T2MI), defined by myocardial necrosis secondary to an imbalance between oxygen supply and demand in the absence of acute atherothrombosis, accounts for a substantial and growing proportion of troponin-positive presentations in clinical practice. Its prevalence has been estimated to range from 10% to over 20% among patients with elevated cardiac troponin concentrations, although this varies substantially depending on patient selection, healthcare setting, and diagnostic criteria application. The use of high-sensitivity cardiac troponin assays has contributed to the increased detection of T2MI though challenges remain in distinguishing it from other forms of myocardial injury or infarction [8,9,10].

A defining epidemiological feature of T2MI is its predominance among older adults. Across multiple cohort studies, the mean age of patients with T2MI consistently exceeds that of those with type 1 myocardial infarction (T1MI), often by 5–10 years. In one large prospective analysis, the average age of T2MI patients was 75 years, compared to 68 years in T1MI [2]. This age difference reflects not only demographic trends but also the pathophysiological vulnerability of older individuals to ischemia triggered by systemic stressors.

Alongside age, multimorbidity is a hallmark of the T2MI population. Patients frequently present with a high burden of chronic conditions, including hypertension, chronic kidney disease, anemia, diabetes mellitus, atrial fibrillation, prior cerebrovascular disease, and congestive heart failure.

These comorbidities not only predispose to the development of T2MI but also contribute significantly to its complex clinical course and high mortality. Data from observational studies have shown that patients with clinical presentation of T2MI are more likely to be women. The most frequent concomitant diagnoses at clinical presentation are cardiac arrhythmias, heart failure, respiratory tract infections or trauma such as bone fractures [2,11]. What often makes this class of patients more challenging from a therapeutic point of view is the concomitant coexistence of a high ischemic and hemorrhagic risk.

While T2MI is often conceptualized as an ischemic event occurring in structurally normal coronary arteries, growing evidence challenges this assumption. Numerous studies have demonstrated that a significant proportion of T2MI patients have underlying coronary artery disease (CAD), often multivessel in nature. Angiographic data suggest that 36% to 78% of T2MI cases are associated with obstructive CAD, particularly among those who undergo invasive assessment [12,13,14]. In one well-characterized cohort, 42% of T2MI patients had a prior diagnosis of ischemic heart disease, and CAD emerged as a strong independent predictor of future cardiovascular events, including recurrent infarction and cardiovascular death [2].

The main issue is how to properly diagnose this entity, manage, and treat these patients with several comorbidities and usually with multivessel disease (MVD).

The presence of multivessel CAD is not merely an incidental finding but carries substantial prognostic weight. Despite the fact that non-cardiovascular causes (such as infections, malignancy, and progressive organ failure) account for the majority of deaths in T2MI patients, the rate of major adverse cardiovascular events (MACEs) remains high and is comparable in crude terms to that observed in T1MI. Importantly, the coexistence of CAD identifies a subgroup of T2MI patients with residual ischemic risk, who may benefit from targeted cardiovascular prevention strategies—although they are often undertreated in routine practice [2,15].

Indeed, underutilization of secondary prevention therapies in T2MI patients with CAD is a recurring observation across studies. Compared with T1MI, these patients are less likely to be discharged with aspirin, statins, or renin–angiotensin system inhibitors, despite having comparable or even higher long-term cardiovascular risk profiles [2,16]. This disparity reflects both diagnostic uncertainty and the absence of dedicated guideline recommendations for this patient group.

In summary, T2MI affects a predominantly elderly and comorbid population, in which multivessel coronary artery disease is common and prognostically relevant. These epidemiological features distinguish T2MI from T1MI and underscore the need for personalized risk stratification, particularly regarding the presence and burden of CAD. Recognition of this phenotype has important implications for both prognosis and therapeutic decision-making.

## 3. Pathophysiological Framework of Type 2 Myocardial Infarction

Type 1 myocardial infarction (T1MI) is classically characterized by an acute coronary syndrome resulting from the rupture or erosion of an atherosclerotic plaque, with subsequent formation of a thrombus that occludes the coronary artery, leading to myocardial ischemia and necrosis. In contrast, type 2 myocardial infarction (T2MI) is defined by the Fourth Universal Definition of Myocardial Infarction (2018) as a condition of myocardial injury with necrosis secondary to a mismatch between myocardial oxygen supply and demand, in the absence of acute atherothrombotic coronary artery obstruction [8].

**Figure 1:**
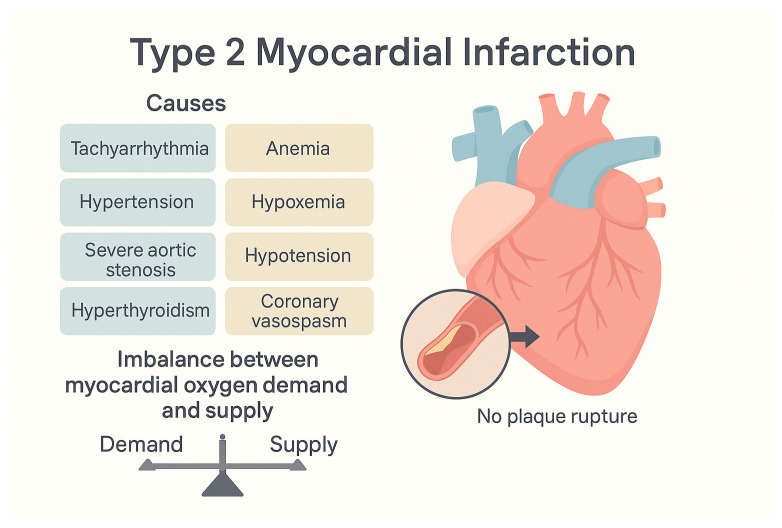
Main precipitating factors in T2MI leading to an imbalance between myocardial oxygen supply and demand.

There are three majors underlying mechanisms of T2MI [2,15] (Figure 1):

- Oxygen demand increases, e.g., tachycardia, hypertension acute volume or pressure overload, hypertensive crisis, or catecholaminergic surge (as in pheochromocytoma or cocaine use) [17].
- Decreased oxygen supply (e.g., anemia, hypoxia, hypotension) due to fixed coronary stenosis.
- Coronary flow is reduced without plaque rupture (e.g., coronary spasm, embolism or microvascular dysfunction).

Furthermore, in certain patient populations, such as the elderly with multivessel CAD or patients with critical aortic stenosis, even minor imbalances in hemodynamic stability can tip the myocardial oxygen supply–demand ratio toward ischemia. On the other end of the spectrum, T2MI may also occur in younger individuals with structurally normal coronary arteries, such as in cases of spontaneous coronary artery dissection (SCAD) or coronary vasospasm, which represent dynamic obstructions without atherosclerotic rupture [9].

From a pathophysiological standpoint, these mechanisms do not lead to transmural infarction in most cases, but rather to subendocardial ischemia, which is more susceptible to oxygen deprivation. Cardiac troponin elevation is a hallmark in T2MI, though it often shows a slower rise and lower peak levels compared to T1MI, reflecting a different ischemic burden and timing [10]. However, troponin kinetics alone are not sufficient for diagnostic distinction and must be interpreted in the clinical context.

As mentioned before, the presence of underlying coronary artery disease is common in T2MI, with some studies reporting angiographic evidence of obstructive CAD in over 40–70% of cases [12]. This raises the question of whether the ischemic burden is exclusively secondary or if there is a contributory role of fixed stenoses under stress conditions—an issue central to ongoing discussions about the appropriateness and timing of invasive evaluation in T2MI.

In case of absence of CAD, T2MI needs to be differentiated from other causes of Myocardial ischemia injury like Angina with Non-Obstructive Coronary Arteries (ANOCA) and Myocardial Infarction with Non-Obstructive Coronary Arteries (MINOCA).

Finally, microvascular dysfunction and endothelial dysfunction may play an important role in the pathophysiology. Endothelial dysfunction primarily reflects impaired nitric-oxide bioavailability, heightened oxidative stress, and a shift toward a pro-inflammatory and pro-thrombotic phenotype, ultimately reducing vasodilatory capacity [18]. In contrast, microvascular dysfunction encompasses structural remodeling, rarefaction, and impaired flow regulation within the small-resistance vessels, which may occur even when large-artery endothelial function appears preserved [19]. Nevertheless, both conditions converge on shared pathophysiological mechanisms, including chronic inflammation, impaired shear-stress signaling, and endothelial–perivascular crosstalk. Clinically, this overlap contributes to heterogeneous presentations ranging from reduced coronary flow reserve to exercise intolerance and heart failure with preserved ejection fraction. Furthermore, systemic endothelial injury can precipitate widespread microvascular dysfunction, underscoring their interdependence.

Thus, T2MI should not be regarded as a benign or self-limiting condition. Its prognostic implications are significant, with high rates of all-cause mortality, often driven by the underlying comorbidities and the triggering systemic illness rather than by the ischemic event itself. This mandates a careful and individualized approach to diagnosis, management, and risk stratification.

## 4. Diagnosis Tools and Current Challenges

### 4.1. High Sensitive Biomarkers

High-sensitivity cardiac troponin (hs-cTn) is the cornerstone biomarker for diagnosing T2MI, but its interpretation requires caution. Absolute values alone do not discriminate between T2MI, T1MI, or non-ischemic myocardial injury, since elevations also occur in conditions such as sepsis, renal dysfunction, pulmonary embolism, or tachyarrhythmia. Serial testing with assessment of the rise and/or fall pattern and correlation with clinical and electrocardiographic evidence of ischemia are therefore essential to establish an acute event [2,8,18].

Moreover, contemporary studies underscore the critical importance of applying sex-specific diagnostic thresholds for hs-cTn I and T, because women often have lower absolute troponin values for a comparable myocardial injury, and adopting uniform cut-offs may lead to underdiagnosis in female patients [20].

In patients with T2MI, the kinetics of hs-cTn differ characteristically from those of T1MI. In T1MI, plaque rupture and acute coronary thrombosis typically produce a rapid and pronounced troponin rise, often with a steep ascending phase and higher peak concentrations that correlate with the extent of necrosis. By contrast, T2MI is usually associated with a more modest elevation, with smaller peak values and a slower or blunted rise/fall pattern, reflecting a mismatch between oxygen supply and demand rather than a large, abrupt occlusion [8,21,22]. Importantly, although the delta change (absolute and relative difference between baseline and repeat hs-cTn) is often smaller in T2MI, there can be overlap with T1MI, especially in patients with severe comorbidities or critical illness. Therefore, reliance on biomarker kinetics alone is insufficient; interpretation requires integration with the clinical setting, electrocardiographic findings, and imaging evidence of ischemia [23,24]. In this context, dual-marker strategies combining hs-cTn with stress-responsive biomarkers such as copeptin have recently been proposed; meta-analyses suggest that the addition of copeptin may improve early sensitivity and negative predictive value, particularly to rule out non-ST-elevation MI in low-to-intermediate risk patients at presentation (e.g., negative predictive value up to ~99.4%) [25].

Although creatine kinase-MB (CK-MB) was historically used in MI diagnosis, it offers no additional diagnostic value over hs-cTn in modern practice and is less sensitive and less specific for myocardial necrosis. Consequently, CK-MB is not recommended for distinguishing T2MI from T1MI or other causes of myocardial injury [8,23].

Copeptin, a surrogate marker of arginine vasopressin release, rises rapidly in acute stress states and may complement hs-cTn for early discrimination. Heart-type fatty acid-binding protein (H-FABP) and myeloperoxidase (MPO) reflect early myocardial injury and inflammatory activation, respectively, and have shown potential in identifying plaque instability characteristic of T1MI. Conversely, markers of systemic inflammation (e.g., C-reactive protein, interleukin-6) and hemodynamic stress (e.g., B-type natriuretic peptide [BNP] or NT-proBNP) are often more elevated in T2MI. However, no single biomarker currently provides sufficient diagnostic accuracy, and integrated multimarker approaches combined with clinical and imaging data are under active investigation to improve differentiation between T1MI and T2MI [26,27,28].

Therefore, further research should focus on validating multimarker panels (e.g., hs-cTn + copeptin) in diverse patient populations, and defining sex-specific hs-cTn cut-offs to optimize diagnostic accuracy and avoid under-recognition, especially in women.

In Table 1 you can find the main features helping to distinguish T1MI from T2MI.

### 4.2. Imaging Diagnostic Tools

Imaging plays a central role in differentiating T2MI from alternative diagnoses. Transthoracic echocardiography may reveal new regional wall-motion abnormalities or conditions causing increased afterload, such as severe aortic stenosis. Invasive coronary angiography is indicated in cases with high suspicion of obstructive coronary artery disease, primarily to rule out a culprit thrombotic lesion consistent with T1MI. Coronary CT angiography (CCTA) provides a non-invasive alternative for excluding significant coronary stenosis in stable patients, particularly when the pre-test probability of coronary disease is low-to-intermediate. Cardiac magnetic resonance (CMR), using the updated 2018 Lake Louise criteria, allows tissue characterization and is highly valuable when the diagnosis remains uncertain: it distinguishes ischemic from non-ischemic injury by identifying patterns of late gadolinium enhancement (subendocardial/transmural vs. mid-wall/epicardial) and detecting myocardial edema. Despite the significant advantages that cardiac magnetic resonance imaging could offer in this setting, its use remains often limited, due to the reduced availability of the technique [23,24,29,30]

**Figure 2:**
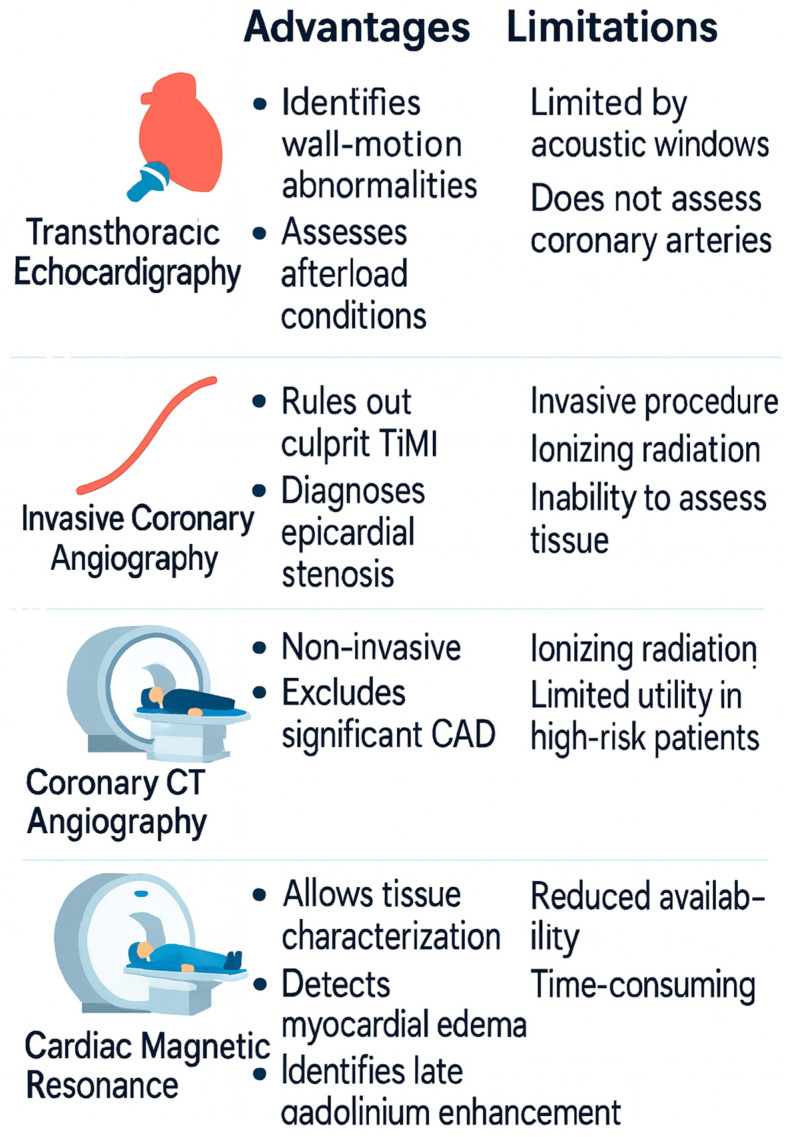
Main advantages and limitations of diagnostic modalities in T2MI.

Finally, T2MI must be distinguished from MINOCA, myocarditis, and atypical T1MI. MINOCA requires fulfillment of the UDMI with coronary stenosis <50% and systematic investigation (often including CMR and intravascular imaging) to identify ischemic vs. non-ischemic mechanisms [31,32]. Myocarditis diagnosis is supported by CMR with the 2018 Lake Louise Criteria Atypical T1MI may lack classic symptoms but usually shows culprit atherothrombosis on angiography or intravascular imaging; its presence mandates reclassification as T1MI [8,24] (Figure 2).

## 5. Therapeutic Approaches

The management of T2MI primarily focuses on identifying and treating the underlying trigger responsible for the oxygen supply–demand imbalance. Correcting precipitating factors such as sepsis, arrhythmias, anemia, or hypoxia is the cornerstone of therapy. Once the acute trigger has been addressed, medical treatment should be tailored to the individual patient’s clinical profile.

### 5.1. Medical Treatment

In T2MI, anti-ischemic measures (e.g., beta-blockers, nitrates, and optimization of hemodynamic) are often considered to reduce myocardial oxygen demand and improve perfusion. Although data are limited, observational cohorts suggest that β-blocker treatment may be associated with better cardiovascular outcomes in some T2MI patients (e.g., lower mortality) [33,34]. However, rigorous randomized evidence is lacking, and dose titration must consider hypotension or conduction abnormalities. T2MI frequently develops in patients with underlying cardiac dysfunction, particularly heart failure (HF), where increased wall stress and impaired coronary perfusion lead to oxygen supply–demand mismatch [33]. Sodium–glucose cotransporter 2 inhibitors (SGLT2i), originally developed for diabetes management, have demonstrated significant cardioprotective effects independent of glycemic control, including reduction in HF hospitalizations, improvement in ventricular remodeling, and attenuation of ischemia–reperfusion injury.

Recent data from the EMPACT-MI trial, which randomized 6522 post-MI patients to empagliflozin versus placebo initiated within 14 days of acute MI, showed a 23% relative risk reduction in first hospitalization for HF (HR 0.77; 95% CI 0.60–0.98; *p* = 0.031) and a 33% reduction in total HF hospitalizations compared with placebo [35]. Similarly, in the DAPA-MI trial (n = 4017), dapagliflozin initiated early post-MI was associated with a favorable hierarchical composite endpoint (win ratio 1.34; 95% CI 1.20–1.50; *p* < 0.001), mostly driven by reductions in new-onset type-2 diabetes, weight loss, and symptomatic improvement rather than direct HF or mortality benefit [36].

In the context of T2MI, SGLT2i may mitigate the hemodynamic and metabolic contributors to myocardial oxygen imbalance by promoting osmotic diuresis, lowering preload and afterload, improving myocardial energetics, and reducing systemic inflammation [35,36,37,38]. Although definitive evidence specifically in T2MI is still limited, these preliminary findings support a potential role of SGLT2i in post-ischemic myocardial protection, particularly in patients at high risk for HF, and underscore the need for dedicated randomized studies focusing on T2MI populations.

The use of antithrombotic therapy in T2MI is particularly controversial. In classical Type 1 MI, dual antiplatelet therapy (DAPT) (aspirin plus a P2Y_12_ inhibitor) is foundational. However, in T2MI the pathophysiology often lacks acute plaque rupture and thrombosis, raising doubts about the benefit of DAPT in all patients. Meta-analyses highlight that DAPT is not routinely justified in T2MI and may increase bleeding risk without clear ischemic benefit [39]. Some observational data in T2MI patients with respiratory failure suggest a possible short-term prognostic benefit of antiplatelet therapy, but residual bias cannot be excluded [40]. Regarding full anticoagulation (e.g., with low molecular weight heparin or direct oral anticoagulants), the evidence is even sparser; a feasibility trial investigating low-dose anticoagulation in T2MI failed to reach recruitment targets, illustrating the challenges of performing trials in this population [41].

Statin therapy, by virtue of plaque stabilization, anti-inflammatory effects, and improvement in endothelial function, is often considered in T2MI patients—especially those with concomitant atherosclerotic disease. Some registry studies suggest that discharge prescription of statins in T2MI is associated with reduced major adverse cardiovascular events, albeit in observational settings [33,34].

#### Ischemic and Hemorrhagic Risk Evaluation

A key challenge in T2MI management is the assessment of bleeding risk versus ischemic benefit, given that these patients often carry comorbidities (e.g., renal dysfunction, anemia, frailty) that predispose to bleeding. In the absence of strong trial data, clinicians must individualize decisions, often leaning toward more conservative antithrombotic strategies when bleeding risk is high.

**Figure 3:**
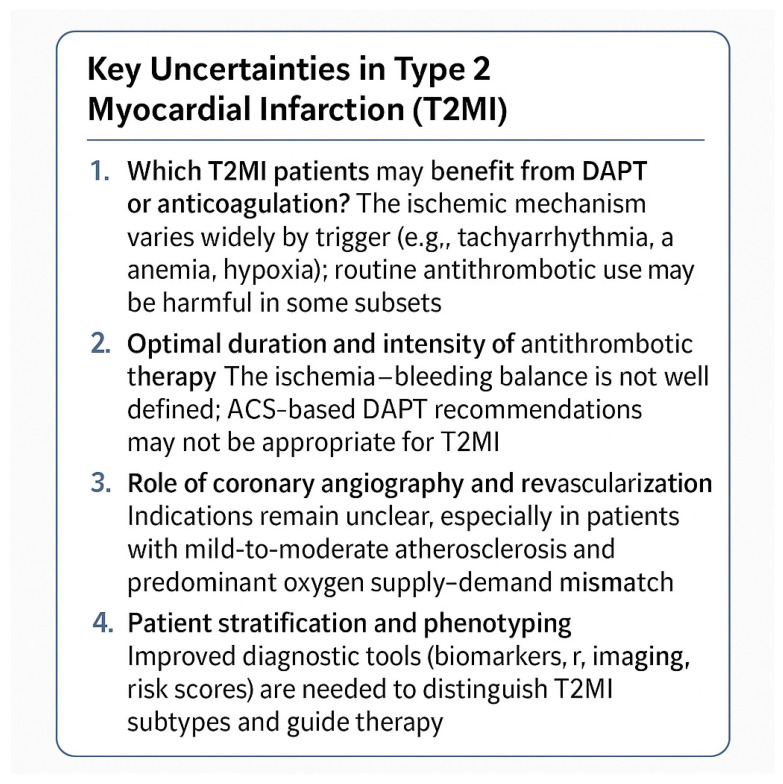

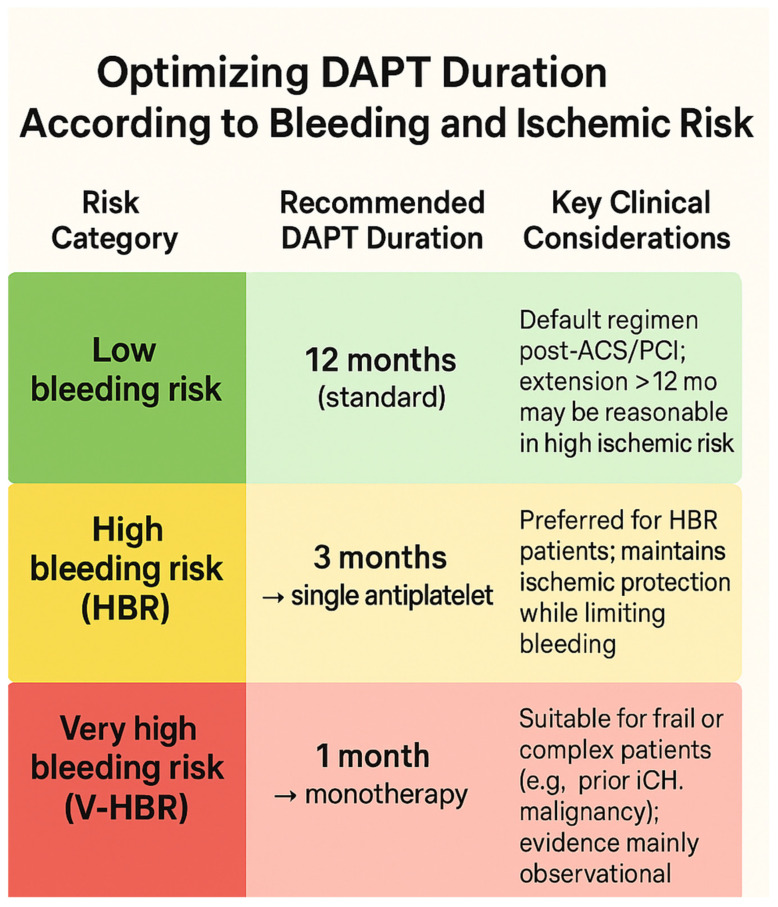
Optimizing DAPT duration related to bleeding risk. ACS (acute coronary syndrome); PCI (percutaneous coronary intervention); HBR (high bleeding risk); V-HBR (very high bleeding risk); iCH (intracranial hemorrhage).

In the general acute coronary syndrome (ACS) literature, high bleeding risk is associated with increased mortality, stroke, and recurrent MI over time [42]. Although T2MI patients are underrepresented in ACS trials, this principle remains relevant: in patients with high bleeding risk, limiting the duration or intensity of antiplatelet therapy may be appropriate. Recent ACS guidelines (e.g., ACC/AHA 2025) emphasize tailoring DAPT duration and selecting agents with favorable safety profiles, but these guidelines are derived primarily from Type 1 MI populations [5,43] (Figure 3).

### 5.2. Role of Coronary Angiography in Type 2 Myocardial Infarct

Coronary angiography and revascularization in case of T2MI remain a topic largely unknown. In type 1 AMI an invasive strategy has largely demonstrated to reduce mortality. Coronary angiography is highly sensitive in identifying the presence of ulcerated and/or thrombotic coronary plaque and is often crucial to differentiate diagnosis, identify thrombosis, and rule out CAD.

On the other hand, the T2MI definition does not take into account the presence of thrombus and can also occur in the absence of coronary artery disease (vasospasm, spelling out SCAD, endothelial dysfunction); despite that, the finding of coronary artery disease in patients with type 2MI is not uncommon, especially in the elderly population. Observational cohort studies report that up to 50–75% of patients with T2MI who undergo coronary angiography exhibit obstructive CAD [10,44,45]. Similar findings come from the APACE registry and the CASABLANCA (Catheter Sampled Blood Archive in Cardiovascular Diseases) study, where obstructive disease was found in 60% of T2MI patients undergoing angiography, with multivessel involvement in nearly half of those cases [13,46].

Clinical selection for invasive assessment remains a challenge. And the decision to proceed with coronary angiography must be weighed in relation to the extent of the ischemic picture and balancing the risks.

Beyond mere detection of CAD, angiographic evaluation plays a relevant diagnostic value. In a retrospective angiographic analysis of patients diagnosed with T2MI, nearly one-third showed evidence of plaque rupture or intracoronary thrombus, prompting a reclassification toward T1MI [46]. This underscores how clinical assessment alone is frequently insufficient to reliably differentiate T2MI from atypical presentations of T1MI, especially in the elderly or those with baseline ECG abnormalities.

Predictors of significant CAD at angiography include typical ischemic symptoms, dynamic ST-T changes, regional wall motion abnormalities on echocardiography, and high baseline cardiovascular risk (e.g., diabetes, CKD, history of stroke) [47]. The combination of elevated troponin levels with disproportionate magnitude relative to the presumed trigger may also raise suspicion of underlying culprit lesions or type 1 mechanisms.

In patients with unclear ischemic burden, particularly when invasive angiography is not immediately indicated due to frailty or comorbidities, non-invasive imaging tools such as coronary computed tomography angiography (CCTA) ± functional flow reserve coronary tomography (FFRCT) offer a valuable alternative. These modalities allow for quantification of anatomical and functional lesion severity and may identify patients who benefit from intensified preventive therapy or deferred revascularization. The DEFINE Type 2 MI study used CCTA and FFRCT to evaluate stable T2MI patients; 92% had identifiable plaque, and 26% had hemodynamically significant stenoses, further reinforcing the high prevalence of subclinical or silent CAD in this population [48]. Notably, most of these patients had no prior history of CAD, suggesting that T2MI may unmask clinically silent coronary disease.

Regarding the balance between risk and benefit, several observational studies show controversial findings for the benefit and the use of coronary angiography. A US-based analysis of over 40,000 T2MI admissions reported that only 13% underwent coronary angiography, with considerable inter-hospital variation [49]. Predictors of non-use included advanced age, female sex, and comorbidity burden, yet paradoxically, these same factors are often associated with worse outcomes in T2MI.

Despite the limited use of an invasive strategy, the performance of coronary angiography has been associated with more favorable outcomes. Several registries, including SWEDEHEART and MINAP, showed that invasive management was associated with reduced all-cause mortality [44,45].

The SENIOR-ITA study, a prospective observational multicenter registry conducted in Italy, provide insight into the role of coronary angiography in elderly patients with acute myocardial infarction, including those classified as T2MI. The study enrolled patients aged ≥75 years presenting with non-ST elevation myocardial infarction (NSTEMI), with a median age of 82 years. Among this population, those who underwent coronary angiography had significantly lower 1-year mortality compared to those managed conservatively (15.9% vs. 30.8%; *p* < 0.001), despite being frailer and having a higher burden of comorbidities. The benefit persisted after adjustment for baseline characteristics, suggesting that an invasive strategy may confer prognostic benefit even in very elderly patients, particularly when selection is based on clinical judgment and not age alone [33].

These findings support a selective but not age-restrictive approach to coronary angiography in older patients with suspected ischemia, including those with T2MI when other high-risk features are present.

In addition, angiographic findings in T2MI are often ambiguous: several studies have demonstrated that up to one-third of T2MI patients show no clear culprit lesion on angiography, and in many cases, ischemia results from dynamic or microvascular mechanisms rather than focal epicardial obstruction [10,12]. This diagnostic uncertainty highlights the importance of adjunctive imaging modalities such as intravascular ultrasound (IVUS) and optical coherence tomography (OCT), which allow detailed assessment of plaque morphology, thrombus, and vessel remodeling that may not be evident on angiography alone.

Coronary angiography in T2MI should be reserved for patients in whom the clinical picture suggests a possible benefit—either for diagnosis clarification, identification of treatable disease, or initiation of secondary prevention.

We suggest a multiparametric approach to help who could benefit from an invasive strategy and when to perform:

- Patients with clinical, ECG, or echocardiographic signs of ischemia.
- Those with known or suspected CAD and recurrent ischemic symptoms.
- Cases with disproportionate troponin elevation or without an obvious extra-cardiac trigger.
- Individuals at high ischemic risk, including diabetics, those with peripheral arterial disease, or severe left ventricular dysfunction.

In this population, a personalized risk-based strategy with invasive coronary angiography, ideally supported by imaging, is preferable to a blanket conservative approach.

## 6. Coronary Imaging and Physiological Assessment to Guide Treatment Strategy in T2MI

In patients with T2MI and multivessel coronary disease, it is often difficult to define whether a specific lesion contributed directly to the ischemic event. In the absence of a clear culprit, intermediate stenoses in multivessel territories pose a clinical dilemma: whether to revascularize or defer treatment. In this setting, the use of intracoronary imaging techniques such as optical coherence tomography (OCT) and intravascular ultrasound (IVUS), along with functional assessments like fractional flow reserve (FFR) and instantaneous wave-free ratio (iFR), can guide personalized decisions between interventional and conservative strategies.

IVUS and OCT enable precise anatomical characterization of lesions, and evidence on the efficacy of the use of imaging techniques is widely documented in the literature in a wide heterogeneity of clinical conditions. The ESC guidelines strongly suggest the use of imaging techniques to guide and optimize coronary angioplasty procedures (class I level of evidence B). In T2MI patients with angiographic ambiguity, these tools can reveal features of plaque vulnerability—such as rupture, erosion, or thrombus—that may not be apparent on angiography alone.

The RENOVATE-Complex PCI trial, a large-scale observational study of patients undergoing PCI for complex coronary anatomy, demonstrated that IVUS-guided PCI was associated with a significant reduction in all-cause mortality compared to angiography-guided procedures (adjusted HR 0.58; 95% CI 0.44–0.75; *p* < 0.001) [50]. Although the primary benefit was observed across multiple endpoints, the reduction in mortality was particularly compelling and consistent across subgroups, including those with bifurcations, long lesions, and left main disease [51]. The advantage of IVUS in this setting lies not only in procedural optimization, but also in accurate identification of the ischemia-driving lesion, a challenge often encountered in T2MI patients where supply–demand mismatch may coexist with silent multivessel coronary artery disease.

A series of meta-analyses published between 2020 and 2023 have further reinforced this survival benefit. A comprehensive 2022 meta-analysis of over 30,000 patients by Ahmed et al. reported that IVUS-guided PCI resulted in a 36% relative risk reduction in all-cause mortality compared to angiography guidance alone (RR 0.64; 95% CI 0.55–0.75) [52]. Other pooled analyses confirmed consistent findings across various subgroups, suggesting that IVUS use is independently associated with improved survival, irrespective of lesion complexity or clinical presentation [53]. These results suggest a class effect of intravascular imaging in improving both short- and long-term outcomes, likely due to more precise stent sizing, expansion, and avoidance of under-treatment of high-risk plaques.

Data supporting the use of OCT comes from the ILUMIEN IV and OPTIMAL PCI trial, which compared OCT-guided stent implantation with angiography-guided PCI in patients with stable or unstable CAD, demonstrating improved procedural outcomes with OCT without an increase in complications [54]. The OCTOBER trial, focused on complex bifurcation lesions, showed that OCT-guided PCI significantly improved stent expansion and procedural optimization compared to standard angiography [55]. Additionally, the OCTIVUS trial directly compared OCT-guided versus IVUS-guided PCI in complex coronary lesions, showing non-inferiority of OCT in terms of target lesion failure and procedural success [56].

Although most evidence derives from ACS cohorts, intravascular imaging has also shown utility in the MINOCA population, where OCT reclassified the underlying mechanism in a substantial proportion of cases, revealing previously undetected plaque disruption or intracoronary thrombus [57].

In patients with suspected spontaneous coronary artery dissection (SCAD) or unusual angiographic patterns, OCT has proven especially helpful in defining dissection planes, intramural hematomas, and distinguishing these from thrombotic or calcified plaques [58]. These data, though limited in T2MI-specific cohorts, support the use of imaging when revascularization is being considered but culprit identification is uncertain.

Functional assessment with FFR or iFR is particularly relevant in T2MI with stable coronary anatomy and no dynamic ECG changes. In such cases, the ischemic burden may be due to systemic stressors on top of fixed obstructive lesions. A prospective study by Weerts et al. in patients with NSTEMI—including a substantial subset of T2MI—demonstrated that FFR guidance safely deferred PCI in non-flow-limiting lesions, with significantly fewer events in the defer group at both 1 and 3 years [59]. Similar findings were reported in studies using non-invasive FFR derived from coronary CT (FFR_CT_), where among patients with ≥50% angiographic stenosis, FFR_CT_ showed that only 26% had hemodynamically significant lesions, and 38% of those with apparent obstruction were reclassified as functionally non-significant [48]. These findings underscore the importance of physiological testing in avoiding unnecessary interventions in T2MI.

In patients with multivessel CAD and no evident culprit lesion, functional testing may help stratify risk and guide which lesions to treat. If a lesion shows FFR ≤ 0.80 or iFR ≤ 0.89, it may be reasonable to proceed with PCI, especially in proximal vessels or in the setting of residual angina. Conversely, in hemodynamically insignificant lesions, optimal medical therapy should be preferred to reduce bleeding risk and procedural complications—particularly in frail or high-bleeding-risk individuals.

In the SCAD population, intracoronary imaging is useful primarily to confirm the diagnosis when doubt persists. However, the management remains mostly conservative. In a systematic review, PCI in SCAD was associated with higher rates of technical failure and repeat revascularization, with no survival benefit over conservative treatment [60]. Imaging here is valuable to support conservative decision-making and to assist in PCI planning when strictly needed.

In clinical practice, intracoronary imaging and physiological tools should be integrated in T2MI when there is

- Multivessel CAD with an uncertain culprit;
- Intermediate lesions without clear ischemic territory;
- A need to limit procedural burden in high-risk patients;
- Diagnostic uncertainty in non-obstructive presentations such as MINOCA or SCAD.

This approach allows safe deferral of PCI in non-flow-limiting lesions and a more tailored strategy in anatomically complex but clinically ambiguous situations, a frequent scenario in the T2MI population.

## 7. Revascularization Strategies: PCI vs. Conservative Treatment

The decision to perform coronary angiography and percutaneous coronary intervention (PCI) in patients with type 2 myocardial infarction (T2MI) must be carefully individualized, considering not only the coronary anatomy but also the systemic condition, comorbidity burden, bleeding risk, and procedural tolerance. While in type 1 MI the benefit of stenting is well established, in T2MI the pathophysiological mechanism is not primarily driven by acute plaque rupture, and therefore the role of stenting is more nuanced.

The revascularization strategy in patients with T2MI is challenged by several concurrent elements: the clinical heterogeneity, the frequent presence of multivessel coronary artery disease (CAD), and the high prevalence of frailty and comorbidities. In observational registries, up to 50–70% of patients with T2MI who underwent angiography exhibited obstructive CAD, often diffuse and calcified, with frequent involvement of the proximal segments or left main [45,61]. However, not all the patients undergo percutaneous coronary intervention (PCI), due to uncertainty in causality between the coronary lesion and the acute event, and the potential risk of harm in this vulnerable population.

PCI should be considered when there is evidence of persistent or inducible ischemia, dynamic ECG changes, or high-risk anatomy, particularly in the presence of symptoms refractory to medical therapy. However, unlike type 1 MI, the benefit of PCI in T2MI has not been demonstrated in randomized trials.

Data from broader populations with stable CAD help contextualize the potential role of PCI in patients with T2MI and extensive disease. The ISCHEMIA trial showed that in patients with stable CAD and moderate to severe ischemia, an initial invasive strategy with PCI or CABG did not reduce major adverse cardiac events (MACEs) compared with optimal medical therapy (OMT), although it did improve angina-related quality of life in symptomatic patients [62]. Similarly, the ORBITA trial, a placebo-controlled study, questioned the symptomatic benefit of PCI in patients with single-vessel stable angina, suggesting a strong placebo component [63].

In patients with ischemic left ventricular dysfunction, the REVIVED-BCIS2 trial showed no reduction in all-cause mortality or heart failure hospitalization with PCI compared to OMT alone, despite extensive CAD and myocardial viability on imaging [64]. These data further question the benefit of revascularization when the ischemic mechanism is not clearly culprit-related or when the primary driver is myocardial dysfunction.

The potential benefit of PCI in T2MI may be more limited in high-risk subsets such as the elderly or those with high bleeding risk (HBR). The MASTER DAPT trial demonstrated that, in HBR patients who underwent PCI, abbreviated dual antiplatelet therapy (DAPT) (1 month followed by monotherapy) was non-inferior to standard therapy for ischemic endpoints and superior in reducing bleeding events [64]. Similarly, a large retrospective registry from MedStar Washington Hospital Center found that in HBR patients with elevated ischemic risk (DAPT score ≥ 2), bleeding events and all-cause mortality were significantly more frequent, with no significant ischemic benefit associated with prolonged DAPT [65]. This underscores the narrow therapeutic window in this population.

One of the recognized limitations in adopting an invasive strategy in patients with type 2 myocardial infarction (T2MI) is the ability to accurately identify the culprit lesion. In this context, the decision to pursue a culprit-only or complete revascularization strategy remains a matter of debate. This issue becomes even more relevant given the high prevalence of multivessel coronary artery disease among patients with T2MI [66].

On one hand, a strategy focused solely on the culprit lesion has the advantage of reducing procedural complexity and duration; on the other hand, identifying the true culprit lesion in this patient population is often challenging, and targeting a single lesion might, in fact, lead to treatment of the wrong vessel.

There are few studies specifically addressing revascularization strategies in patients with NSTEMI and multivessel disease. The limited available evidence suggests a trend toward benefit with a complete revascularization strategy, whether guided by angiography or by physiological assessment. The multicenter randomized FIRE trial (1445 patients aged ≥75 years with both STEMI and NSTEMI) showed that the complete revascularization strategy significantly reduced the composite primary endpoint of all-cause death, myocardial infarction, stroke, or ischemia-driven revascularization at 1-year follow-up (15.7% in the complete revascularization group vs. 21% in the culprit-only group; HR 0.73, 95% CI 0.57–0.93). It should be emphasized that, in this study, the selection of non-culprit lesions was also guided using coronary physiology assessment.

Balancing the risks and benefit from an invasive strategy, a minimal invasive approach could be reasonable in many T2MI patients. This is particularly relevant in patients ≥ 75 years of age, those with CKD, active malignancy, recent bleeding, or on oral anticoagulants. The use of intracoronary imaging (e.g., IVUS, OCT) and physiological assessment (e.g., FFR) may aid in refining the indication for PCI, minimizing unnecessary interventions (Table 2).

When an invasive approach is deemed appropriate in T2MI, the choice of revascularization strategy requires a nuanced assessment of ischemic benefit versus procedural and bleeding risks. These considerations become particularly relevant in subsets such as frail patients, those with high-bleeding-risk criteria, small-vessel or diffuse coronary disease, or complex anatomical patterns. In such scenarios, different invasive options—including conventional DES-based PCI, no-stent strategies, and DCB-based approaches—offer varying profiles of safety and efficacy. In the following chapters, we will examine these strategies in greater detail, discussing their specific risks, benefits, and evidence across distinct clinical settings, thereby providing a practical framework for individualized revascularization planning in T2MI.

## 8. Alternative Strategies to Drug-Eluting Stents (DES)

Standard drug-eluting stents (DESs) remain the cornerstone of percutaneous coronary intervention (PCI), offering a durable mechanical scaffold and effective local drug delivery that significantly reduces restenosis and target lesion failure. Their performance in a wide range of clinical and anatomical settings is well established. However, DES implantation entails the permanent presence of a metallic scaffold, which can impair vasomotion, provoke chronic inflammation, and require dual antiplatelet therapy (DAPT) for at least 1–3 months—factors that may represent substantial limitations in specific high-risk subsets such as elderly, frail, or high-bleeding-risk (HBR) patients, and in those with diabetes mellitus or multivessel disease (MVD).

DESs ensure immediate luminal gain and predictable outcomes, but at the cost of vessel caging, potential neoatherosclerosis, and a residual risk of late stent thrombosis. In contrast, stent-free approaches—most notably drug-coated balloons (DCBs)—enable antiproliferative drug delivery without leaving a permanent implant. This preserves native vessel physiology, reduces chronic inflammatory stimuli, and allows for much shorter DAPT regimens. Such a paradigm shift toward “leave-nothing-behind” strategies has gained considerable traction, particularly in patients for whom prolonged DAPT or additional metallic layers are undesirable.

The BIONICS registry, which included over 2000 patients undergoing PCI with modern DESs for diffuse or small-vessel disease, reported a 12-month target lesion failure (TLF) rate of 5.3%, with diabetes, vessel diameter < 2.5 mm, and lesion length > 20 mm emerging as independent predictors of adverse outcomes [67]. Similarly, the ULTRA registry, focused on complex subsets (left main, bifurcations, and chronic total occlusions), showed clinically relevant residual event rates despite the use of ultrathin-strut DESs, with lesion complexity, renal dysfunction, and ACS presentation being key determinants [68]. These features frequently coexist in patients with type 2 myocardial infarction (T2MI).

Moreover, elderly and frail T2MI patients often have concomitant anemia, renal dysfunction, or active bleeding, placing them in the HBR category. In these individuals, the use of DES mandates at least short-term DAPT, which—even when abbreviated—can be poorly tolerated. The MASTER DAPT trial demonstrated that a 1-month DAPT regimen after biodegradable-polymer DES implantation was non-inferior to longer therapy for ischemic protection while reducing major bleeding [65]. Yet even abbreviated or de-escalated regimens may remain unsafe in patients with recent gastrointestinal bleeding, thrombocytopenia, or those requiring oral anticoagulation. Procedural complications—such as vascular access bleeding, contrast nephropathy, and peri-procedural myocardial injury—are also more frequent in this frail population.

**Figure 4:**
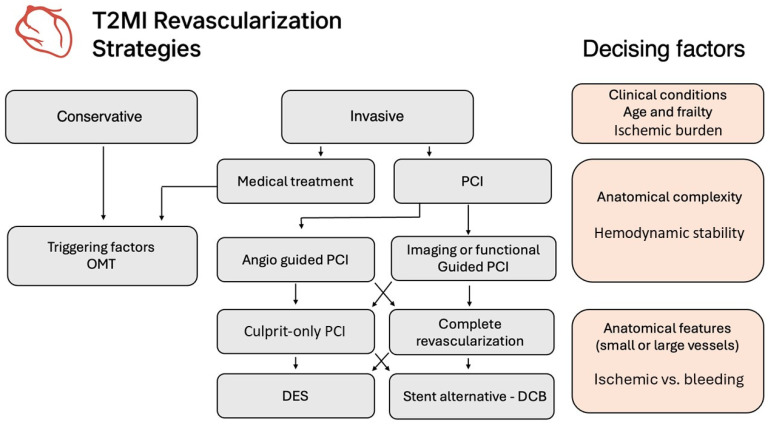
Therapeutic flowchart in T2MI related to revascularization strategies. OMT (optimal medical therapy); PCI (percutaneous coronary intervention); DCB (drug coated balloon).

These limitations have rekindled interest in stent-free strategies such as DCB angioplasty or even “no-stent” PCI, which aim to minimize the metallic footprint and reduce the dependency on prolonged DAPT (Figure 4).

## 9. Drug-Coated Balloons (DCBs): Mechanism and Rationale

DCBs deliver an antiproliferative drug—most commonly paclitaxel or sirolimus—directly into the vessel wall via a short balloon inflation, ensuring homogeneous local drug transfer without the implantation of a permanent scaffold. This approach eliminates chronic mechanical irritation, preserves vasomotion, and allows DAPT duration to be minimized, often to 1 month.

According to the recent Drug-Coated Balloon Academic Research Consortium (ARC) Consensus Document (Fezzi et al., JACC 2025) [69], DCBs offer several intrinsic advantages over DES:

1. Homogeneous drug delivery to the vessel wall.
2. Absence of a permanent implant, allowing for late positive remodeling and preserved vasoreactivity.
3. Possibility of reducing DAPT duration and intensity.
4. Avoidance of side-branch jailing and carina shift in bifurcations.
5. No interference with future interventions or imaging.
6. Feasibility for combination (“hybrid”) strategies.
7. Applicability to challenging anatomies (diffuse disease, small vessels, bifurcations, MVD) and high-risk clinical subsets (HBR, diabetes, ACS, elderly).

Historically, DCBs were validated in the treatment of in-stent restenosis (ISR), for which they remain the preferred therapy (Class I, Level A recommendation in European guidelines) [70]. The field has since expanded toward de novo lesions, particularly in small vessels and high-risk patients.

The PICCOLETO II and BASKET-SMALL 2 trials both demonstrated the non-inferiority of DCBs versus DESs in small-vessel disease, with DCBs providing comparable angiographic and clinical outcomes, and the additional benefit of reduced late lumen loss [71,72,73]. The DEBUT trial, which enrolled high bleeding risk patients—including those with ACS—showed the superiority of DCB over bare-metal stents (BMSs), with markedly lower major adverse cardiac event (MACE) rates at 9 months (1% vs. 14%) [67]. Similarly, the BELLO trial compared paclitaxel-coated balloon angioplasty with everolimus-eluting DES in small vessels, demonstrating comparable MACE at 12 months (7.5% vs. 7.9%) and highlighting the advantage of shorter DAPT [74].

In diabetic and multivessel patients, a large observational study by Lee et al. [75] compared DCB-based strategies (standalone or hybrid DCB + focal DES) with DES-only PCI in over 1100 diabetic patients. Outcomes at 2 years were similar in terms of target vessel failure, cardiac death, and myocardial infarction, while DCB-based interventions resulted in fewer target lesion revascularizations and less late stent thrombosis.

Recent meta-analyses have reinforced the non-inferiority of DCBs compared with DES in small-vessel disease, diabetic patients, and high-bleeding-risk individuals. Verdoia et al. confirmed similar late lumen loss and MACE rates between DCBs and DESs in diabetic and HBR populations, while Somsen et al. emphasized the procedural simplicity and safety of DCBs in frail patients undergoing complex PCI [76,77].

Despite the growing body of evidence supporting DCB use across a range of anatomies and clinical settings, currently there are still some important limitations regarding the use of DCBs. Randomized data remain largely confined to patients with small-vessel disease, ISR, or HBR presentations, whereas evidence in complex lesions (bifurcations, diffuse disease, ostial lesions) is still evolving. In addition to trials data, technical success remains dependent on achieving an optimal predilation result—residual stenosis < 30% and absence of major dissections—which may not always be attainable in heavily calcified or tortuous vessels. In this view, the operator’s experience and adherence to lesion preparation protocols critically influence outcomes, representing a source of variability not fully accounted for in current studies. Patient’s selection is equally crucial. While DCBs may be particularly appealing in frail, elderly, or HBR patients, as well as in small-vessel or diffuse disease, diabetic and multivessel patients could benefit from hybrid approaches (combined CABG and PCI), although the evidence is still predominantly observational. Finally, no trial has been specifically designed for the T2MI population. Existing trials and registries primarily involve T1MI or stable CAD, with only limited inclusion of ACS patients and virtually no stratification by myocardial infarction subtype. Therefore, while DCBs may theoretically provide advantages in T2MI—particularly by shortening DAPT exposure and avoiding stent implantation—their adoption in this population relies on extrapolation from broader high-risk groups rather than direct evidence. This gap highlights the need for dedicated studies evaluating DCBs in T2MI, especially given the unique interplay of comorbidity, bleeding risk, and coronary anatomy typical of this entity.

## 10. Bleeding Risk: Insights from the 2025 ARC Consensus Statement

The 2025 JACC ARC Position Paper represents the most comprehensive expert consensus on DCBs to date [69]. The document emphasizes that the absence of a permanent metallic scaffold may be especially advantageous in HBR, diabetic, and multivessel patients—conditions in which DES efficacy is often challenged by anatomical and systemic comorbidities. The consensus supports a “DCB-first” or “hybrid” (DCB + DES) approach for appropriate de novo lesions and complex anatomies, particularly when minimizing total stent length is desirable.

The success of DCB therapy, however, is highly dependent on optimal lesion preparation, including adequate predilatation, <30% residual stenosis, and absence of major dissection. The consensus strongly encourages the use of intravascular imaging (IVUS or OCT) to guide vessel sizing, plaque assessment, and post-procedural evaluation. When properly executed, DCB-only PCI can achieve favorable long-term outcomes while substantially reducing metallic burden and the need for prolonged DAPT.

## 11. The “No-Stent” and Deferred-Stenting Strategies

Minimalist revascularization strategies—such as deferred stenting or balloon angioplasty alone (POBA)—may be appropriate in selected scenarios with preserved flow. In the DANAMI-3-DEFER trial, patients with STEMI and restored TIMI 3 flow after initial balloon inflation had similar outcomes to those undergoing immediate DES implantation [78]. Although extrapolation to T2MI requires caution, these findings suggest that, in select lesions with adequate preparation and no flow-limiting residual stenosis, permanent stenting may not be necessary, thereby reducing both procedural and pharmacologic risks.

## 12. Antiplatelet Therapy in Type 2 Myocardial Infarction: Balancing Ischemic and Bleeding Risk

In patients with type 2 myocardial infarction (T2MI), the routine use of dual antiplatelet therapy (DAPT) remains controversial, as most presentations are not linked to plaque rupture or thrombus formation. Nonetheless, a considerable number of T2MI patients have coexistent coronary artery disease (CAD) or undergo percutaneous coronary intervention (PCI), where a tailored antiplatelet strategy becomes clinically relevant.

Importantly, no major randomized trial has been specifically designed for the T2MI population, and current antiplatelet recommendations are largely extrapolated from studies conducted in T1MI or mixed ACS cohorts.

According to the 2023 ESC ACS guidelines, DAPT remains the default approach for 12 months following ACS and PCI (Class I, level A), irrespective of stent type. However, in specific populations—such as those meeting high-bleeding-risk (HBR) criteria (e.g., ARC-HBR or PRECISE-DAPT ≥ 25)—a 1-month DAPT followed by single antiplatelet therapy (SAPT) is considered acceptable (Class IIb, level B). In non-HBR patients without high ischemic risk, 3- or 6-month DAPT durations may be considered (Class IIa, level A), depending on the individual profile [66].

This concept of treatment individualization has gained support from a growing body of randomized data and meta-analyses, which increasingly challenge the 12-month “default” strategy. The SIDNEY-2 meta-analysis, involving over 24,000 patients undergoing PCI, showed that a short DAPT (1–3 months) followed by P2Y_12_ inhibitor monotherapy did not increase ischemic events (MI, stroke, death) and significantly halved major bleeding (BARC 3 or 5), both in HBR and non-HBR patients [66,79]. Notably, ticagrelor monotherapy showed a mortality benefit compared to continued DAPT, whereas clopidogrel did not.

Similarly, the T-PASS trial demonstrated that ticagrelor monotherapy after only 16 days of DAPT was non-inferior—and even superior—to a full 12-month DAPT regimen, with a significant reduction in major bleeding without increasing ischemic outcomes [75]. Conversely, the STOP-DAPT 2 ACS trial failed to demonstrate non-inferiority of short DAPT followed by clopidogrel monotherapy, emphasizing the importance of drug selection, particularly in higher-risk presentations [79].

Among HBR patients, the MASTER-DAPT trial (n > 4400) showed that 1-month DAPT followed by monotherapy was non-inferior to longer DAPT (≥3 months) in terms of net adverse events (death, MI, stroke, or major bleeding) [65]. A companion meta-analysis of 11 trials (>9000 HBR patients) reinforced this finding, showing that abbreviated DAPT regimens reduced cardiovascular mortality and bleeding, without increasing ischemic complications [80].

Parallel to the interest in DAPT shortening, a strategy of DAPT de-escalation has been studied. Switching from potent P2Y_12_ inhibitors (ticagrelor or prasugrel) to clopidogrel or reducing the dose of ticagrelor (from 90 to 60 mg bid) aims to maintain anti-ischemic protection while minimizing bleeding. A different meta-analysis showed that de-escalation significantly reduces both bleeding and ischemic events compared to standard DAPT [81].

Despite these findings, current ESC guidelines maintain a conservative stance: 1-month DAPT followed by SAPT remains only Class IIb (for HBR patients), while 3- and 6-month strategies hold a IIa recommendation. As highlighted in recent critical appraisals, this may represent a missed opportunity to align guideline recommendations with contemporary trial evidence [66] (Table 3).

## 13. Future Perspectives

Despite recent advances in the understanding of type 2 myocardial infarction (T2MI), significant gaps remain in both diagnosis and management. Current definitions are largely conceptual and fail to fully capture the heterogeneity of this condition. The absence of validated diagnostic algorithms and dedicated therapeutic trials limits the translation of evidence into clinical practice.

Future research should prioritize the development of integrated diagnostic frameworks that combine clinical assessment, biomarker profiling, and advanced imaging to improve diagnostic precision. Prospective studies are needed to define which subsets of T2MI patients may benefit from invasive evaluation and tailored revascularization, particularly in the context of multivessel disease or recurrent ischemia.

Moreover, the inclusion of T2MI patients in ongoing and future randomized interventional trials are essential to clarify the role of PCI, drug-coated balloon strategies, and optimal antithrombotic regimens in this fragile population. Finally, machine learning-based risk stratification models and multimodal registries may help refine individualized management and guide real-world clinical decisions.

## 14. Conclusions

Type 2 myocardial infarction represents a growing clinical challenge at the intersection between acute coronary syndromes and systemic disease. Its complex pathophysiology, overlapping presentations, and high burden of comorbidity require a multidisciplinary and individualized approach.

While invasive evaluation and revascularization may improve outcomes in selected high-risk patients, a conservative or alternative strategy—supported by physiological and imaging guidance—should be considered in the elderly and high-bleeding-risk populations.

Ultimately, the optimal management of T2MI lies in accurate diagnosis, refined ischemic risk assessment, and tailored therapeutic decisions that balance ischemic benefit against procedural and bleeding risk. Ongoing efforts to standardize diagnostic criteria and integrate personalized interventional approaches will be crucial to improving outcomes in this heterogeneous and often underrecognized patient group.

## Figures and Tables

**Table 1:** Comparison of type 1 myocardial infarction (T1MI), type 2 myocardial infarction (T2MI), and myocardial injury (Non-MI) with the relatives pathological mechanisms (arrows).

	T1MI → Plaque Rupture/Thrombosis	T2MI → O_2_ Imbalance	Non-MI → Non-Ischemic Injury
Primary Mechanism	Atherothrombosis → ↓ Flow	O_2_ supply < demand	Direct injury (myocarditis, sepsis)
Troponin Kinetics	Rapid rise → Fall	Mild/Delayed rise → Fall	Stable or minimal change
ECG Pattern	ST-elevation/ischemic changes	Variable/nonspecific	Usually normal
Coronary Findings	Obstructive CAD (plaque rupture)	Non-obstructive or normal	Normal coronary arteries
Clinical Context	Acute coronary event	Supply–demand mismatch (e.g., anemia, tachyarrhythmia)	Inflammatory/toxic/stress related injury

**Table 2:** DAPT, double antiplatelet therapy; NSTEMI, non-ST elevation myocardial infarction; PCI, percutaneous coronary intervention; STEMI, ST elevation myocardial infarction.

Strategy	Pros	Cons
Standard Stenting (DES)	− Robust evidence in STEMI/NSTEMI − Enables full revascularization	− Prolonged DAPT increase bleeding risk (HBR, elderly) − Risk of stent thrombosis if DAPT interrupted − Procedural burden in frail patients
Drug-Coated Balloon (DCB)	− No permanent implant − 1-month DAPT feasible − Avoids late stent issues	− Limited MI data − Mostly studied in small vessels/stable lesions − Risk of recoil/dissection; bail-out stent may be needed
No-Stent PCI	− Ultra-short DAPT possible − Comparable outcomes in selected STEMI cases	− Risk of recoil/restenosis/closure − Sparse data beyond niche settings − Higher re-intervention risk

**Table 3:** Tailored antiplatelet therapy strategies in ACS/T2MI context. Table shows the antiplatelet regimen suggested for the different clinical setting and its evidence according to ESC guidelines. T2MI (type 2 myocardial infarction); CAD (coronary artery disease); ACS (acute coronary syndrome); SAPT (single antiplatelet therapy); DAPT (dual antiplatelet therapy); OCT (optical coherence tomography); OAC (oral anti-coagulant); TAT (Triple antiplatelet therapy). Arrows for the suggested therapy strategy.

Clinical Setting	Antiplatelet Strategy	ESC 2023 Class/LoE
T2MI without CAD or PCI	No routine antiplatelet therapy; case-by-case if no evidence of CAD/ACS	No specific recommendation (expert judgment)
Obstructive CAD, medically managed (ACS)	Default: DAPT up to 12 months → Consider SAPT at 3–6 months if event-free and not high ischemic risk (prefer P2Y_12_)	I (default DAPT); IIa A for SAPT at 3–6 mo.
PCI + High Bleeding Risk (HBR)	Abbreviated DAPT 1 month → SAPT (often clopidogrel)	IIb B/A
PCI + Low Bleeding Risk (ACS)	Default: DAPT 12 months; consider SAPT 3–6 months if event free or low risk	I (12 mo); IIa A (SAPT 3–6 mo)
Suspected plaque rupture (e.g., OCT-confirmed ACS)	Standard 12-month DAPT	I (guideline default)
DAPT de-escalation (e.g., ticagrelor → clopidogrel)	May be considered to reduce bleeding after the first month; avoid <30 days	IIb A (consider); III B (avoid <30 d)
Patients ≥70 years with ACS	Clopidogrel may be considered (vs potent P2Y_12_) in elderly/HBR	IIb B (POPULAR-AGE)
ACS + long-term OAC indication	TAT up to 1 week → OAC + SAPT up to 12 mo → OAC alone (>12 mo). OAC alone can be considered 6–12 mo in selected (IIb)	I (early TAT then OAC + SAPT); IIb (OAC alone 6–12 mo)

## Data Availability

No new data were created or analyzed in this study.
